# Supplementary material for: High-Power Ultrasound in Gas Phase: Effects on the Bioactive Compounds Release from Red Bell Pepper during In Vitro Gastrointestinal Digestion
Source: Antioxidants (Basel). 2023 Feb 2;12(2):356. doi: 10.3390/antiox12020356 (PMC9952216; doi:10.3390/antiox12020356)
Supplement: Supplementary file 1 [file antioxidants-12-00356-s001.zip › antioxidants-2163687-supplementary.pdf]

Table S1. Moisture content, total polyphenols content (TPC) and antioxidant activity (AA) before and after simulated *in vitro* digestion in both the solid and the juice (J). Different letters for the same parameter indicate significant differences ($p<0.05$) between samples.

	Time (min)	Moisture	FRAP	CUPRAC	ABTS	TPC
**Red bell pepper**	0	11.66 ± 1.06 b	27.82 ± 0.30 b	32.60 ± 1.11 a	32.94 ± 1.45 a	14.48 ± 0.30 b
	120	7.75 ± 0.60 c	35.18 ± 1.07 a	34.08 ± 0.65 a	34.33 ± 0.63 a	19.62 ± 1.50 a
**Gastric sample**	0	15.79 ± 0.46 a	26.92 ± 0.50 b	28.87 ± 0.61 b	23.51 ± 1.19 c	13.48 ± 0.33 bc
	120	13.65 ± 0.51 b	24.52 ± 0.17 c	26.00 ± 1.70 bc	20.65 ± 1.67 c	12.32 ± 0.52 c
**Gastric phase**	0	-	3.25 ± 0.19 g	4.79 ± 0.97 e	9.88 ± 0.94 e	5.40 ± 0.45 e
	120	-	12.59 ± 1.12 f	10.55 ± 1.05 d	16.33 ± 0.17 d	9.23 ± 0.35 d
**Intestinal sample**	0	13.64 ± 0.53 b	20.65 ± 1.22 d	27.29 ± 2.73 bc	35.04 ± 1.51 a	13.67 ± 0.55 bc
	120	13.36 ± 1.01 b	16.10 ± 0.76 e	23.91 ± 0.59 c	29.14 ± 2.69 b	12.73 ± 0.28 c
**Intestinal phase**	0	-	0.85 ± 0.07 h	4.38 ± 0.25 e	8.45 ± 0.63 e	2.48 ± 0.40 f
	120	-	1.93 ± 0.22 gh	5.75 ± 0.21 e	10.46 ± 0.42 e	4.36 ± 0.14 e
